# Institutional shared resources and translational cancer research

**DOI:** 10.1186/1479-5876-7-54

**Published:** 2009-06-29

**Authors:** Paolo De Paoli

**Affiliations:** 1 Centro di Riferimento Oncologico, IRCCS, Via F Gallini, 2, I-33081 Aviano PN Aviano, Italy

## Abstract

The development and maintenance of adequate shared infrastructures is considered a major goal for academic centers promoting translational research programs. Among infrastructures favoring translational research, centralized facilities characterized by shared, multidisciplinary use of expensive laboratory instrumentation, or by complex computer hardware and software and/or by high professional skills are necessary to maintain or improve institutional scientific competitiveness. The success or failure of a shared resource program also depends on the choice of appropriate institutional policies and requires an effective institutional governance regarding decisions on staffing, existence and composition of advisory committees, policies and of defined mechanisms of reporting, budgeting and financial support of each resource. Shared Resources represent a widely diffused model to sustain cancer research; in fact, web sites from an impressive number of research Institutes and Universities in the U.S. contain pages dedicated to the SR that have been established in each Center, making a complete view of the situation impossible. However, a nation-wide overview of how Cancer Centers develop SR programs is available on the web site for NCI-designated Cancer Centers in the U.S., while in Europe, information is available for individual Cancer centers. This article will briefly summarize the institutional policies, the organizational needs, the characteristics, scientific aims, and future developments of SRs necessary to develop effective translational research programs in oncology.

In fact, the physical build-up of SRs per se is not sufficient for the successful translation of biomedical research. Appropriate policies to improve the academic culture in collaboration, the availability of educational programs for translational investigators, the existence of administrative facilitations for translational research and an efficient organization supporting clinical trial recruitment and management represent essential tools, providing solutions to overcome existing barriers in the development of translational research in biomedical research centers.

## Introduction

In the last few years there has been a tremendous expansion in translational research studies requiring integrated multidisciplinary efforts or special expertise that are not widely available to individual researchers. In fact, single laboratories, clinical divisions, or research groups do not possess sufficient financial funding, space or well-trained personnel to afford such opportunities. Therefore, the development and maintenance of adequate shared infrastructures is considered a major goal for academic centers promoting translational research programs [1,2]. Among infrastructures favoring translational research, centralized facilities characterized by shared, multidisciplinary use (by different departments, Divisions, Research Units) of expensive laboratory instrumentation, or by complex computer hardware and software and/or by high professional skills are necessary to maintain or improve institutional scientific competitiveness. This article may be particularly interesting for the scientific community since it includes the novel, exhaustive analysis of the shared resources necessary to support research activities in a comprehensive cancer center. Aims and advantages of establishing efficient shared resources for research centers and for investigators can be summarized as follows [3,4]:

- Institutional, rather than individual, investments offer the opportunity to buy the most technically advanced, high throughput instrumentation to be used by each research group.

- Single researchers may have access to new methods or to a multiparametric characterization of tumor models by the use of several technologies contained in the whole set of SRs present in the Institute, an approach that is generally much more cost effective than establishing the technique in each research group laboratory.

- Availability to all researchers of highly trained personnel with specialized skills in the technologies present in the Institute.

- Given the rapid evolution of biomedical research and technologies, the continuous users' education is an important issue. The availability of highly trained staff in each SR technology permits the provision of an advanced education and training programs to all other investigators.

- Quality control programs based on extensive expertise of the users, appropriate setting of the instruments, may lead to superior experimental results because of increased sensitivity, accuracy, and reproducibility.

- The presence of highly advanced SRs usually results in an increase of interdisciplinary collaborations and enhancement of translational research programs.

- Centralized purchase procedures invariably result in reduction of reagent costs, maintenance of equipment, and personnel expenditure.

### Establishing SRs or outsourcing services

In order to fulfill the need of new technologies in support of innovative fields within biomedical research, a institution may consider establishing a new SR instead of simply outsourcing its services, based on several aspects: cost effectiveness, turnaround time, flexibility of services offered, commercial availability, and technical quality of the data. All these tasks are equally important since, for example, some technical services may be quite expensive, but commercially unavailable because of the high level of technical expertise required or inconvenience of marketing due to insufficient numbers of researchers who are interested in using particular techniques. On the contrary, outsourcing may be convenient when economically advantageous for the institution or when the half- life of a technology is too short or uncertain to deserve a financial investment. Decisions regarding the technologies to outsource, selection of partners, and the management of such relationships are of crucial importance for institutions aiming at developing competitive research programs. This process may be accomplished through the establishment of criteria, for example through a Decision Support Framework model containing a set of guidelines and procedures useful for Institutional executives to effectively manage decisions on whether to source technologies internally or externally [5]. Biomedical research increasingly depends on very sophisticated resources or on interdisciplinary collaboration that may be not adequately satisfied by simply outsourcing technologies or services. In these cases the creation of shared resources consortia including several institutions [6,7] or of national or international infrastructure programs may be necessary to adequately develop biomedical research programs [8,9]. As an example, the European Roadmap for Research Infrastructure is based on the construction and operation of a consortium including governmental and scientific partners from several European countries [9].

### Helpful and harmful policies

The success or failure of a shared resource program also depends on the choice of appropriate institutional policies. Due to the importance of this issue, policies fostering or disregarding the establishment and appropriate functioning of SRs have been identified both in literature as well as in day-to-day practice in many Institutes [4]. Although generally applicable policies on resource sharing are not possible due to differences in the resources to be shared, the needs of SR users and the type of research programs to be developed in each institution [10] are suggested as useful for stimulating the use of SR:

- The presence and amount of institutional funds that partially share the cost of SR encourage their use by scientists, especially by young researchers who may not yet have fully established laboratory equipment and personnel.

- The redistribution of obtained economies to develop new research programs, buy new technologies or hire personnel with higher qualifications supporting cross- sectional institutional research activities reinforces the perception of the importance of having efficient shared infrastructures.

- Academic long term commitment for upgrading space, instrumentation, staff training, and financial support; this commitment may be practically realized through the appointment of a qualified Director of all the SR in the institution, who chairs an Advisory Committee that meets regularly to review the information regarding the usage, performance, and customer satisfaction of the SRs and the availability and performance of new technologies present in the market. Based on the Committee's suggestions, the Cancer Center leadership may implement the SR program.

- Promote knowledge of the available technologies by including a period of training in SR in educational programs for graduate and post-doctoral students increases their use by more research groups.

- Greater emphasis on scientific opportunities and advantages for the entire scientific staff, and scientific excellence may stimulate a positive loop resulting in increased scientific productivity.

- Project planning of SRs includes clear guidelines about ownership and access to SRs and about property and scientific use of the data obtained from SR activities; furthermore, the ability to guarantee equitable access to all researchers interested in SR use is mandatory. These are essential ingredients in preventing later misunderstandings and problems.

- While the above-mentioned options may improve the successful establishment of SRs, problems may arise when harmful policies are applied. A few examples of harmful policies may be:

- Lack of incentives to share resources could result in conflicts and academic staff frustration; institutions lacking an environment that facilitates sharing of productive ideas and resources among investigators from different disciplines may experience requests of unnecessary duplication of instrumentation, staff, and expertise by single researchers and incapacity to access high value technology. Ultimately, this leads to the difficulty in developing successful translational research programs.

- Lack of professional opportunities for SR personnel also, negatively affects the presence of high quality SRs. In fact, the success of SR depends upon the attraction of high scientific level staff. The opportunity to develop scientific research of top quality by using sophisticated technologies and the interaction with top level scientists who are part of an academic center's staff, may be key factors in attracting skilled managers and technicians devoted to SR functioning.

- Lack of sufficient financial support. The research centers developing an SR program must be aware that the purchase and maintenance of technology equipment is very costly; accordingly, the availability of excellent SR staffs requires salaries and benefits adequate to their professional skills.

- Although the establishment of SRs requires substantial financial investments, overemphasis on costs savings rather than on the benefits that inevitably result in productivity and excellence of research programs is probably considered the policy that mostly damages the development of SRs [4].

### Governance

The appropriate maintenance and development of shared resources requires an effective institutional governance regarding decisions on staffing, existence, and composition of advisory committees, policies, and defined mechanisms of reporting, budgeting, and financial support of each resource.

#### Staffing

As previously mentioned, the presence of a high quality staff is an essential component in developing a SR system in Cancer Centers and in other research Institutions. Depending on the characteristics of each SR, the staff must be composed of peculiar professional profiles; the responsibilities of the staff encompass several activities, extending beyond technical and educational skills, such as planning and problem solving, communication skills, and the ability to share research programs and experimental results with other scientists. Generally speaking, the role of staff could be:

- To prepare a user guide defining general policies, services provided, sample preparation and fees; planning (reservations) and performing experiments.

- The use and maintenance of the instrumentation, including troubleshooting problems.

- To define and program the acquisition of reagents and supplies for daily operational procedures, according to the SR assigned budget.

- To set up new methods and technologies that are strongly requested by research groups in the Cancer Center.

- To establish a productive communication with each research group discussing experimental design and results as well as collaborating in preparing grant proposals or scientific manuscripts.

- To evaluate new instruments on the market and contribute to the long term strategies of the SR by sending suggestions to the SR committees.

SR staff may be constituted by the Director/Medical Director, Administrative Director, the Facility Manager and by a member of technical staff. The Facility Manager provides, in consultation with the SR manager and the users or advisory committees, when present, strategic suggestions to the Board of Directors to establish or modify policy issues, plans and establishes the budget; he/she also proposes acquisition of new instruments and interacts with Cancer Center leadership on program issues. He/she may provide consultation for grant application and preparation of scientific reports. The Manager is usually an internal researcher of the institution who has special expertise in the field devoting a variable percentage of his/her activity to oversee the entire operational aspects of the SR.

The Facility Manager is the first point of contact for many prospective users of the facility and is responsible for the daily operations of the SR, including work scheduling, supervision of staff, service and maintenance of the equipment and training programs; she/he assesses each user's research needs, suggests effective experimental approaches and recommends protocols as necessary to obtain the data needed. In addition she/he may be involved in the development of protocols, consultation on experimental design, analysis and interpretation.

Depending on the operational needs and on the complexity of the technologies included in the SR, the staff includes a variable number of laboratory technicians, biostatisticians, biomedical engineers, nurses, and data managers. The Facility Manager and the technicians are usually fully devoted to develop SR activities.

#### Advisory committees

Committees may also be essential components of the SRs. An appropriate users' committee may be appointed for each SR that periodically evaluates the performance, the utilization, and the costs/productivity of the SR. Furthermore, each committee may assess future needs for technological, financial, and human resources of the SR and prepare a proposal to be evaluated by the SR director and, eventually, by an oversight committee. The overall activity and the strategic value for the Center of all the SRs available may be assessed by a SR Oversight Institutional Committee including core managers, directors of research programs, a director of the administration; this committee interacts with the Directorate of the Institute to discuss the development of an institutional SR program, including the development or discontinuation of individual SRs, the contract of resources, services proposed for the future, and the impact of SR on institutional research programs or the overall impact of SRs on research goals of the Institute.

The appointment of an External Advisory Committee may be necessary for SRs requiring very high technology investments or having nation-wide or international usage; this committee could support institutional decisions on the purchase of equipment or on the establishment of relationships with international partners, pharmaceutical, and biotechnological industries.

### Policies

Access policies include the modality of SRs use. Scheduling may be planned on first come-first served basis via web-based systems or paper registries. The involvement of personnel in assisting individual users may vary: assisted use means that users require the assistance of a technician from the SR, this may also signify that users who plan the experiments and/or prepare the samples, while running the instrumentation, rely partially or completely on SR staff. In unassisted use, sample preparation, use of the instrumentation, and interpretation of results relies completely on single investigators and the role of the SR consists in providing efficient instrumentation and in running quality controls. In fact, those users who completed the training and demonstrated the ability to use the equipment without technical support may be certified as independent users, which provides them the opportunity to independently use the equipment, including during off-peak hours. Due to technical complexity, some SRs may function only through assisted use.

Usage policies include the fees for each, assisted or unassisted, procedure that are established by the Institution depending on the calculated costs of the SR (space, instrumentation, personnel), on the cost of reagents, on the usage frequency by individual research groups within the Institution or by external users and on the availability of an institutional support budget that may be assigned annually to the functioning of SRs. In the U.S., part of the costs of institutional SRs can be requested through NCI Cancer-Center Support Grants [11].

Policies also include the rules governing intellectual property of experimental results and their relevance for the development of research projects and grants. The degree of involvement by facility staff in planning, execution, and discussion of each project depends on the nature and difficulty of the project and also on the prior expertise of the investigators in that field.

#### Periodical reporting systems, budgeting and financial support of SR

SRs are usually maintained by institutional funds and users fees. The latter may support a portion of daily operational costs, while institutional support is mandatory to cover additional costs; in particular, the purchase of new equipment and the development of new technologies. Finally, while each SR functions independently, a very important task is to create a unifying information and tracking system to integrate all the data present in the SRs of each institution. This integration will allow the Cancer Center Board of Directors to efficiently develop annual budgeting issues as well as mid-term strategic plans.

### Examples of existing shared resources in cancer centers

Shared resources represent a widely diffuse model to sustain cancer research; in fact, web sites from an impressive number of research Institutes and Universities in the U.S. contain pages dedicated to SRs that have been established in each Center, making a complete view of the situation impossible. However, a nation-wide overview of how Cancer Centers develop SR programs is available for the NCI-designated Cancer Centers in the U.S. [11]. In European countries, information on institutional SR is usually limited to the situation present in each Center; however, the European Community has recently developed centralized technological platforms that may constitute a trans-national model of integration [9].

According to the NCI Cancer Center Overview on shared resources, January 2008 update, the majority of Cancer Centers possess at least the following shared resources: Flow Cytometry, Genomics (or DNA sequencing, microarray, etc), Proteomics, Animal Facilities (in more than 50% of Institutes there is a distinct additional Genetically Engineered Mouse facility), Biostatistics, Bioinformatics, and Clinical Research Office. The type of additional, less represented, Shared Resources is quite heterogeneous and depends on the scientific orientation of each Center (i.e. more clinical or basic research oriented). Some of the more diffused or more relevant SRs for translational cancer research programs are included in the following list:

- Confocal Microscopy

- Flow Cytometry

- Genomics or DNA sequencing, microarray, cytogenetics

- Proteomics

- Pathology

- Animal facilities, including imaging, genetically engineered mice

- Biobanking/Tumor bank

- Bioinformatics

- Biostatistics

- Pharmacology

- Clinical Research Office

However, there is increasing evidence supporting the observation that advances in basic science do not always result in direct benefits for patients by their incorporation in standard medical practices; although the reasons for such failures are multiple and complex, probably one of the most important obstacles is the consistent observation that results obtained in animal models often do not apply to humans [12,13]. In order to overcome these problems, new types of centralized facilities have recently been developed; these facilities are not based, as most of the above mentioned SRs, on technologies but rather on complementary innovative approaches owed to the measure of specific functions, like the immunological response, or testing novel treatment modalities, for example in radiation therapy, or to specifically promote translational research programs. I have selected the following as examples of these types of innovative SRs:

- Human immunologic Monitoring

- Radiation Resources

- Translational research

In the following paragraphs, the institutional policies, organizational needs, characteristics, scientific aims, and future developments of SRs necessary to develop effective translational research programs in oncology, will be briefly summarized.

### Confocal microscopy

The high resolution imaging of subcellular components, specific proteins, and other biological molecules represents a very important opportunity in cancer research. Conventional optical microscopy enables a two-dimensional evaluation of biological specimens, while the material is organized in three dimensions. Confocal microscopy permits collection of three-dimensional images from living or fixed cells and tissues by the use of laser scanning technology. This technique has gained popularity in biomedical cancer research [14] and has allowed for analysis of several processes of tumorigenesis, such as angiogenesis and its inhibition by biological molecules [15,16], the expression and regulation of cellular receptors involved in cancer development [17], the interaction of oncogenes in control of DNA replication and cancerogenesis [18].

The primary characteristics of CM arise from the use of a pinhole to prevent out of focus light that may degrade the image; this system detects only the light within the focal plane, eliminating the background caused by out-of focus light and scatter from images and producing a higher resolution as compared to conventional optical microscopy; in addition, CM permits the acquisition of serial images from living cells on timescales from milliseconds to hours. Biological laser scanning confocal microscopy is almost invariably associated with fluorescent probes that specifically target subcellular components, such as nuclei, mitochondria or the cytoskeleton, even cellular processes, such as apoptosis, enzymatic activities, etc. Therefore, a complete confocal microscopy apparatus consists of the optical microscope and a light emitting source such as lasers; the most commonly used lasers include argon-ion with usable power at 257, 477, and 514 nm and helium neon lasers with usable power at 534, 567, and 612 nm [19]. The system also consists of reflecting mirrors, interference filters to select the appropriate light wavelengths, and electronic light detectors (photomultipliers); the detector is attached to a computer which reconstructs the image and permits storage and further analysis of the experimental data. A Confocal Microscopy SR requires space, such as housing in one or two, temperature-controlled, laboratory rooms and financial investments to purchase microscopic equipment and computers; the facility's staff consists of a Director, a manager, and one or more laboratory technicians.

### Flow cytometry

Flow cytometry is a technique used to measure predefined physical and chemical properties of cells or particles suspended in a stream of fluid. This technique was initially developed to characterize and separate a heterogeneous mixture of cells into distinct populations for phenotyping or functional analysis. The modern flow cytometer consists of a light source, usually a laser, optical detectors, electronics, and a computer to translate signals into data. Although flow cytometry may be considered a mature technique, substantial improvements have been made in the last few years [20,21]. For example, older instruments only had a single laser and three or four optical detectors, while newer instruments have up to four lasers and more than 15 detectors, although the majority of flow cytometers employed for research and diagnostics typically measure only 6 to 12 parameters. Recent progress in laser technology permits the sale of machines including light sources emitting at UV (around 355 nm), violet (approx. 405 nm), blue (488 nm), green (approx. 532 nm), red (approx. 635 nm); concomitantly, the development of new fluorochromes and new software tools capable of analyzing large and complex data sets made provision for the set up of a highly complex multiparameter flow cytometry (up to 18 colors plus two physical parameters, cell size and granularity) [20,21]. These measurements are not limited to the phenotypic analysis of cells, but also permit simultaneous measurement of several other biological parameters in living cells, such as the cell cycle or other cellular pathways [22,23]. In particular, flow cytometry can be extremely useful in cancer research by quantifying cellular DNA or RNA content, cellular proliferation, oncogene and tumor antigen expression, and the phosphorylation of signal transducers reflecting the activation of specific cell-signaling networks [24,25].

Multi-parameter flow cytometry is routinely used in diagnostic laboratories to characterize hematopoietic cells for the diagnosis and classification of hematologic tumors, including the detection of minimal residual disease, of immune system diseases, for measuring in vivo and in vitro specific immune response to infectious agents, cancer vaccines, and autoantigens [26-28]. The multi-parameter aspect of flow cytometry is particularly useful in implementing cancer research protocols that study the behavior of single cells included in a heterogeneous mixture of cellular populations, such as those found in tumor samples [25]. Recent studies pointed out the presence, in many cancers, of alterations to genes encoding signaling pathways. Identification of these alterations is important for the development of anticancer therapies, as demonstrated by the tyrosine-kinase inhibitor, Imatinib, successfully used to treat patients with chronic myeloid leukemia [29]. Flow cytometry constitutes an ideal tool to distinguish alterations in specific signaling pathways of single tumor cells, that may be normal in non neoplastic cells, contaminating the tumor samples. In conclusion, the application of flow cytometric techniques to characterize biological aspects of tumor cells and the effects, induced by experimental compounds, on altered signaling pathways is very useful to improve clinical success of anticancer drugs. Less commonly used applications of flow cytometry involve monitoring of fluorescent marker-associated transfection assays and particle-based immunoassays using beads to measure soluble analytes, such as cytokines [30,31]. More recently, microsphere arrays have been used to profile miRNA in cancer cells, providing a new application of the flow cytometric technique [32].

Flow cytometers can be equipped with cell sorting devices. These machines can analyze many fluorescence and physical parameters of individual cells and purify those that meet predefined characteristics, i.e. a certain phenotype or DNA content. Current cell sorters are high-speed cell sorters, separating up to 70,000 events per second [33,34]. Many sorters use a jet-in-air separation, while in other cases a highly sensitive sorting cell flow is used [33]. Cell sorters may be used to study rare events in cells separated from bulky cellular populations, for cell based therapies [34], for sperm sorting, for gender pre-selection [35], for chromosome sorting [36], etc..

### Genomic Technologies

Genomic technologies offer important tools to analyze large numbers of gene structures or regulation by identifying DNA mutations and deletions, by assessing the amounts of RNA present in biological specimens, or by epigenetic or karyotypic analyses [37]. In particular, DNA microarrays are widely used for diagnostics, prognostics and predictions of response to therapy in various cancers [38,39]. Microarray analyses are prone to disruptions (noise, false positives, poor reproducibility) [40,41], however they are technologically evolving and more efficient instruments/technology will be made available commercially [42,43]. These technologies differ in the characteristics of the probes, deposition technology, labeling and hybridizing protocols, possibility for single or multiple fluorophore analyses and cost. The Illumina Technology uses probes adsorbed on silica beads. The recently developed tiling arrays are particularly suitable for identification of unknown transcripts, DNA methylation changes, and DNA-protein interactions [44]. All these approaches have advantages and disadvantages, but the primary factor determining differences in the analytical results is biological rather than technical [42]. In addition, differences in the design of different platforms can facilitate the analysis of different biological parameters or pathways, thus acting as complementary rather than alternative tools. Although in many institutions DNA sequencing services are outsourced, several institutions maintain in-house sequencing services. The standard DNA sequencing apparatus is based on the evolution of the Sanger chemistry technique that has a low throughput (1–2 million base pairs per day) and higher analytic costs, but it offers the advantage of reading long fragments (550–800 bp) and having a very high accuracy [45]. Pyrosequencing is a new DNA sequencing technology based on a different, commercially available, system. As compared to the traditional technique, pyrosequencing offers much higher throughput analysis (200 million bases per day) and a simplified preparation process. Major limitations of this technology include short-read lengths and a reduced sequencing accuracy for some genomic regions. New generation technologies include the Illumina Solexa's genome analyzer, the AB Solid Platform, and the HeliScope sequencer [45,46]. All these technologies offer a high throughput capacity (>200 million base pair per day) at a reasonable cost per analysis. While the Illumina Solexa's has already been introduced on the market, experience with the other two technologies is still limited and their performances remain to be fully established. The choice of purchasing one of the DNA sequencing technologies depends on the workload of the Shared Resource and the cost of the apparatus: ranging from several hundred thousand dollars up to a million dollars [46]. Although first generation technology (that is, Sanger) requires support of additional instrumentations and has a higher cost per analysis, it probably remains the technology of choice for small-scale projects. The important differences existing among second generation technologies (that are, pyrosequencing, Illumina Solexa's, SOLiD, and HeliScope) may result in advantages of one technology compared to the others for specific research projects and applications. In parallel, the success of second generation sequencing instrumentation will require a substantial progress in the development of software and bioinformatics tools for data analysis [46].

Molecular cytogenetic aspects are becoming more important for cancer research projects. Traditionally, cytogenetics refers to the study of the description of chromosome structure and alterations that cause diseases [47]. More recently, molecular techniques were applied to cytogenetics allowing identification of chromosomal abnormalities with high resolution. These methods are particularly important in cancer research and diagnostics as cancer genomes accumulate several genetic and karyotypic abnormalities in regions that harbor tumor suppressor genes or oncogenes. These techniques therefore provide important insights into the molecular mechanisms of cancer generation and progression. Therefore, the development of cytogenetic services is becoming one of the tasks of Genomic SR within cancer research institutes. Molecular cytogenetics is mostly based on fluorescence in situ hybridization (FISH) or chromogen in situ hybridization (CISH). Both techniques require basic laboratory equipment, probe labeling, and hybridization tools. In addition, FISH requires the availability of a fluorescence microscope that may be equipped with systems for a complete imaging analysis of fluorescence signals. For this reason, CISH may be more suitable for a pathology laboratory relying on standard optical microscopes. The assessment of FISH and CISH performances in cancer research and diagnosis is beyond the scope of this article and is described in several excellent reviews [47-49]. In situ hybridization techniques are performed on metaphase chromosomes that could be difficult to prepare, especially in solid tumors, thus limiting their widespread use [47]. Comparative genomic hybridization (CGH) has been developed to overcome this problem [50]. Currently, CGH is coupled to array technology allowing analysis of the whole genome or it may be applied to the analysis of specific genomic regions of interest that may give essential information in particular types of cancers [47,51].

Areas of development in the field of genomics include high throughput analysis of the trascriptome based on the sequencing of a technology that may overcome several problems encountered with the use of microarrays [52,53], ultra deep sequencing platforms.

### Pathology

In some Cancer Centers this resource consists in a standard Pathology laboratory providing routine histology services (such as cutting and staining of fresh or paraffin-embedded tissues to be used for analytical techniques), expert histopathology evaluations, immunohistochemistry, and in situ hybridization techniques for human and experimental tissue samples. In these cases, the SR is organized as a standard Pathology laboratory, including adequate space, safety hoods, equipment for surgical pathology, automated slide-stainers, optical microscopes, and refrigerating/freezing devices, etc.. Several other Centers have organized facilities with aims and services that are more complex or more research- oriented, such as macromolecular services (DNA/RNA isolation, quantitation and distribution) or experimental/research pathology and/or molecular pathology core. Experimental/Molecular Pathology cores use advanced, high throughput techniques for the molecular characterization of tumor cells [54]; in these SRs additional instruments may include automated DNA/RNA extraction systems, centrifuges, instruments for nucleic acid amplification such as thermocyclers or Real Time PCR machines, etc.. Tissue microarray (TMA) represents a high-throughput technology for the assessment of hundreds of samples on a single microscope slide by histology-based tests such as immunohistochemistry and fluorescence in situ-hybridization [55]. TMA technology has been applied to the study of tumor biology, such as the characterization of oncogenes in breast and prostate cancers [56,57], for the assessment of new diagnostic tools, such as protein expression in lymphomas and adenocarcinomas [58,59] and the assessment of prognostic tools, such as in breast cancer [60]. The TMA equipment includes a tissue microarrayer required to remove tissue cores from samples and insert the core into TMA specific blocks. TMA blocks are then stained by immunohistochemistry or fluorescence in-situ hybridization. Scoring of the TMA can be performed under light microscopy or, when available, the TMA can be digitally scanned and displayed on a monitor. Although automated TMA instrumentations have greatly increased standardization and quality control programs, TMA studies still suffer from the same issues that affect traditional whole-section analyses, such as dependence on good quality tissues, validated antibodies, and on an accurate standardization of the technique [55]. The staff of this SR may include pathologists and expert technicians in surgical pathology, histology, immunological, and molecular techniques in oncology. Specific skills are necessary when automated instrumentations are essential parts of the facility.

A common problem encountered by cancer researchers arises from the heterogeneous nature of tumor tissues that may confound molecular analysis. In order to overcome this problem, a novel technique of laser microdissection has been recently developed and microdissection services are currently offered in several advanced pathology SRs. With this technique, cells of interest may be identified via microscopy and then removed from heterogeneous tissue sections via laser energy [61]. Then, purified cells can be further analyzed by DNA genotyping, gene expression analysis at the mRNA level, or by signal-pathway profiling and proteomic analysis [62,63]. Laser microdissection instruments are based on infrared or ultraviolet systems, both in the manual and the automated platform configuration [61]. Presently, a laser microdissection apparatus is seldom present in a pathology SR in cancer institutes, but it may soon become an essential tool for translational research programs in oncology.

In some Institutes, the Tissue Bank facility is included in this SR, but I consider biobanking as a separate entity, one devoted to collection and storage not only of tissues, but also blood, blood products, biological fluids, and nucleic acids as well as maintaining an informatics platform connected with other existing databases (i.e. genomic, proteomic, immunologic, and clinical).

Future developments within this SR may regard the implementation of novel technologies for image analysis of tissues and the development of tissue pharmacodynamic analytical tools that may be of great value in the management of patients included in clinical trials and in evaluation of innovative drug efficacy.

### Proteomics

Proteomics include the detection, identification, and measurement of proteins and/or peptides, protein modifications (i.e. identification of phosphorylation sites), and the study of protein-protein or protein-DNA interactions and regulation. Proteomic application to cancer provides important information on biomarkers for early detection of tumor development, tumor profiling for diagnostic and staging purposes, and on mapping of cancer signaling pathways aimed at developing new treatments [37,64,65]. In some Cancer Research Centers, the Proteomic SRs have alternative names, such as Cancer Proteomics, Mass Spectrometry, Protein Chemistry and/or Protein Expression [11]. Many different technologies have been applied for proteomic profiling of cancer, including two-dimensional gel-electrophoresis, liquid chromatography coupled with mass spectrometry and antibody-based microarray techniques [66-68]. Due to their analytical sensitivity, large dynamic ranges of detection, and relatively high throughput, mass spectrometry instruments are the preferred technology in proteomic SRs. In addition, mass spectrometry offers important advantages on the other proteomic techniques including the reduced size of the required sample, the possibility to provide information on various aspects of protein structure, regulatory mechanisms, and the analysis of complex proteic mixtures such as serum, plasma, or cellular lysates. Mapping the post-translational modification of protein is another scientific goal that can be more appropriately solved by using a different type of proteomic instrumentation, such as the quadrupole linear ion trap mass spectrometer (QTRAP) [69]. In addition to the above-mentioned instruments, a proteomic SR may require investments to buy other technologies, such as antibody-based microarrays, an HPLC system, two dimensional gel electrophoresis systems, robotic stations for sample preparation, small equipment for processing samples (biological hoods, centrifuges), equipment for protein chemistry analysis, and freezers/refrigerators to store samples and reagents.

The relative expression of proteins in biological samples can also be conducted by non-mass spectrometry, non-microarray based platforms. In general, some techniques that are standard in laboratories, such as ELISA or Western blot, can be considered as proteomic platforms. More recently the Luminex's xMAP technology, an innovative multisphere-based multiplexing system, has been used to measure proteins in biological samples because of several advantages as compared to traditional assays. Some examples of its analytical capabilities that can be performed by using small sample volumes are multiparametric analysis of cytokines, of intracellular signaling pathways, and of protein phosphorylation [70,71].

Some proteomic SRs include services for the production of synthetic peptides that are used for the generation of specific antibodies, the preparation of peptide vaccines, as bioactive molecules, etc.. Commercially available peptide synthesizers may be purchased to perform peptide synthesis.

A current problem in proteomic research is the lack of standards allowing comparisons of the analytic performance in different platforms and/or laboratories. For this reason, future goals of proteomic studies require that researchers with documented expertise, such as those included in Institutional proteomic SRs, develop collaborative protocols that, through identification and validation of common sets of standards, may ultimately permit sharing and comparison of analytical results among various research groups.

### Animal facilities

Animal models are widely used in biomedical research to establish new diagnostic and treatment procedures and study basic mechanisms resulting in the development of several diseases. In particular, investigations in animal models are invaluable in discovering new approaches for diagnosis and treatment of cancer in humans. Animal facilities in various Centers have alternative names, including laboratory animal resource, genetically engineered mouse, transgenic mouse, and animal imaging resource [11]. All these animal facilities support animal research activities, providing housing and care to animals, in particular to mice that represent, for their ease of breeding in captivity and biological characteristics, one of the best animal models for cancer research [72]. Basic space requirements for this SR include animal housing rooms, laboratory procedure rooms, cleaning and sanitizing spaces, a veterinary care space, and staff support areas. More sophisticated animal facilities may include a pathology service room, an imaging facility, a genetically engineered animal facility or others.

According to the NIH guide for the Care and Use of laboratory Animals, animal facilities must be designed considering several factors: in particular, the species, strains, and breed of animals and the goals of the research projects conducted at the Institution. Animal facilities must have adequate space, proper conditions of temperature, humidity, ventilation, and illumination. In addition, facilities must include an Institutional Animal Care and Use Committee and adequately trained personnel caring for animals.

Genetically engineered animal SR (also known are genetically engineered mouse or Transgenic mouse facility) may be included in general animal facilities or constitute a separate entity. Genetically engineered mouse models may accurately mimic the pathophysiological and molecular features of human cancers. The purpose of this facility is to provide a service that efficiently produces genetically-engineered mice for basic and translational research, including transgenic and knock-out mice essential to develop animal models for human diseases and study many biological aspects of disease pathogenesis and response to treatments.

So as to promote genetic studies on the nature of human cancers, the mouse genome can be modified by the pronuclear integration of exogenous DNA (transgenic mouse), by blastocyst injection of genetically modified ES (embryonic stem) cells (chimeric mouse) or by the excision (knock-out mouse) or alterations (knock-in) of gene functions [73,74]. This facility may include a laboratory possessing standard equipment required for cell cultures and to conduct the production and in vivo use of gene-targeting constructs (biological safety cabinets, incubators, microinjection apparatus, etc). As an alternative, genetic material for the production of a transgenic mouse can be provided by individual investigators.

Future developments of genetically engineered animal facilities should take into account that new technologies may be developed to overcome actual limitations of current genetic manipulations of experimental animal models [72].

In vivo imaging consists in the use of non-invasive techniques to monitor the tumor development, progression, and effects of therapeutic interventions; animal facilities using miniaturized conventional imaging techniques, such as CT scan or PET have been developed in several institutions. Animal Imaging is seldom, if ever, included in a separate SR, but it is usually included in integrated services offered by animal facilities. Besides the use of traditional imaging techniques, a new modality, combining in vivo imaging techniques and molecular techniques has been recently developed. Molecular imaging permits the non-invasive visualization of cellular processes at a molecular or genetic level by using imaging probes. It offers the possibility to integrate the detection of molecular alterations with anatomical information specific to each animal or patient, when used in human trials. Animal molecular imaging facilities are particularly useful in those institutions pursuing drug development programs. All of the imaging techniques used in cancer patients have been adapted for use in small animals; the most widely used include magnetic resonance imaging (micro-MRI), x-ray computed tomography (micro-CT), and positron emission tomography (Micro-PET), while single photon emission tomography (SPECT), fluorescence imaging, and ultrasound imaging are less useful in cancer research imaging; excellent literature reviews providing detailed information on animal imaging technologies and techniques are available [75-77].

Micro-MRI provides ultra sensitive (around 100 micron) information on tumor or metastasis localizations and, by using contrast agents, information on tumor vascularity. Micro-MRI Spectroscopy can be used to detect individual targets using magnetically-labeled affinity molecules. Major limitations of micro-MRI are the need of high quality personnel training and the costs of the apparatus [77].

The Micro-CT apparatus is also available in animal SRs; it also has an optimal anatomical resolution (around 50 micron) and can be particularly useful to study discrete anatomical sites, such as lung and bone [75,76]. It offers advantages of limited cost of the apparatus, rapid session times, and limited technical skills required for its use and maintenance.

Although the anatomical resolution of Micro-PET in animals is low (in the order of 1–2 mm) the major advantage of this technique is the use of labeled molecules such as fluoro-deoxyglucose (FDG, radioactive fluorine) that are rapidly taken up by tumors and measure cellular metabolism and functions. The cellular targets of labeled probes can be metabolites, antigens, or genes expressed in normal or pathological tissues. Micro-PET can be used to track cell trafficking, tissue hypoxia, DNA proliferation, apoptosis, angiogenesis, etc. Although the anatomical resolution of micro-PET is low, other advantages are the requirement of a medium-level personnel training and affordable costs.

Advantages and limitations of the use of Micro-PET are similar to those identified in human studies and include the possibility of monitoring molecular events early in the course of the disease or during treatments, while limitations include the limited spatial resolution and the short half life of isotopes [78].

SPECT is a special type of CT scan using radioactive tracers that is able to provide high-resolution images and analysis of multiple biological parameters [73]. SPECT-CT fusion imaging offers advantages as a clinical reporter of cell migration especially useful in cancer immunotherapeutic protocols [79].

Ultrasound is a quick and inexpensive technique to screen animals in vivo for tumor development or monitor in vivo interventional procedures [80], but, due to limitations in the information that can be obtained, its use is quite limited as compared to the above-mentioned animal imaging techniques. The cost of establishing a complete animal imaging facility may be quite high, although in perspective, it may permit allocation of extramural grants covering part of the expenses. A critical point is the need of personnel trained both in animal care and imaging techniques.

### Biobanking

Biobanking is an emerging activity that includes the collection and preservation of biological samples (tissues, cells, serum, plasma, and nucleic acids). The collection of human material is situated at the beginning of the chain of translational research and therefore biobanks are actively contributing to advances in translational research by offering opportunities to safely collect and store these samples and link laboratory research to clinical practice, ultimately accelerating the development of personalized medicine [81,82]. Within this context, the tremendous advances recently reached by high throughput "omics" research (genomics, proteomics, transcriptomics) have created an absolute need to design large-scale, multiparametric experimental protocols that are based on repositories containing well-defined biological samples. Although in some institutions the centralized collection system is included within the Pathology SR, the institution of a specific entity devoted exclusively to the collection of tissues, blood, blood products, and other biological specimens (i.e. nucleic acids, microorganisms) may be a very effective way of improving multidisciplinary research projects [83]. According to guidelines published by the International Society for Biological Environmental Repositories (ISBER), the design of biobanking facilities should include sufficient space to accommodate the material to be stored and provide for the safe movement of people, equipment, and specimens [84]. Security systems should include restricted access only to authorized personnel, uninterruptible power supplies for storing devices and a protection system assuring the respect of ethical and legal issues established at national or supranational levels [85,86].

Laboratory space and instrumentation requirements include a processing room with thermostatic incubators, biohazard cabinets, centrifuges, and a personal computer with a dedicated software that allows management of archived samples and related information. Additional rooms host storage facilities, like -20°C and -80°C freezers, liquid nitrogen containers, or +4°C freezers that incorporate remote alarm systems to store specimens. The freezing devices must have enough space and temperature conditions to permit their correct functioning (i.e. insufficient space or high temperatures may cause overheating and damage of the cooling systems); liquid nitrogen tanks ideally should be automatically filled from high volume liquid nitrogen reservoirs. Automation procedures may permit great improvement in biobanking throughputs, quality control, and costs. The early phases of the biobanking process already benefit from these procedures, since automated liquid handling and sample dispensing systems are presently available in several laboratories and biobanking facilities [87]; in the last few years, process control software supporting laboratory hardware has greatly improved the automation of the additional phases of the biobanking process, i.e. the storage and retrieval of samples from biobanks [88,89].

Ideally, the collection of biological specimen process should be linked to the database containing clinical information and a tracking system of stored samples enabling researchers to recover and be aware of the potential development of translational research applications as well as to recover samples needed to develop their projects very rapidly [81]. In this context, it is particularly important to identify samples from patients entering in clinical trials using innovative therapeutic approaches and interface such information with biological and clinical databases.

Although remarkable examples of large-scale international studies based on tumor biobanking already exist [90,91], future developments include the need to promote inter-institutional cooperation between biological banks. ISBER identified two major crucial subjects within this topic: standardization of sample collection/storage procedures and quality control programs to avoid intrinsic bias in multicenter studies [92]. Enabling multicenter studies on national or international levels also requires a definition of common legal issues [89,93].

### Bioinformatics

Bioinformatics is an interdisciplinary field that integrates computer science and biostatistics with biomedical sciences [94]. It emerged as an essential discipline with the development of high throughput genomic technologies a few years ago [95,96]. With the advent of gene expression microarrays, it became very popular to make data publicly available, not only resulting in public databases but also in the development of open source analysis software [53,96,97]. Nowadays, bioinformatics skills are strongly required in those institutions developing research programs based on high throughput technologies that result in the production of large quantities of data, such as genomics and proteomics [98-100]. The Bioinformatics SR provides expertise to biomedical researchers in data analysis and methodologies using state-of-the-art software, databases, and innovative bioinformatics methodologies. Thus, SR may perform research into new methods and new software aiming at analyzing the structure and functions of biological specimens. It can also support centralized, clinical trials computerized systems and provide expertise for the development of software integrating biological and clinical data with patient samples stored in biological banks. In U.S. Cancer Centers, this facility drives the participation of each center to the cancer Biomedical Informatics Grid (CaBIG), an initiative overseen by the National Cancer Institute [101]. This initiative addresses the critical problems related to the explosion of biomedical data requiring new approaches for collection, management, and analysis. In fact, CaBIG consists of interoperable software tools, data standards, and computing infrastructure conceived to advance basic and clinical research. As of May 2008, more than 60 Cancer Centers are in the process of getting connected to CaBIG tools [101].

The Bioinformatics facility requires space to host a high performance computing system for intensive analysis that includes strong data protection security systems. The staff may be composed of computer and bioinformatics specialists with a sufficient background in molecular biology, genetics, physics, or in other biomedical disciplines that constitute part of the research programs in that institution.

Future tasks may regard the development of infrastructure that allows more integration between clinical informatics and bioinformatics itself, leading to more effective programs in cancer biology and therapy.

### Biostatistics

Appropriate statistical methods are necessary throughout the entire translational research process, from in vitro studies to interpretation of genomic and proteomic analyses, validation of biomarkers, clinical trail design, analysis and data reporting [102]. The Biostatistics SR offers the necessary infrastructure, facilitating interactions between researchers and biostatisticians. In fact, this SR may provide expertise to investigators in the design (help to identify outcome variables and covariates in the choice of appropriate study design, calculate required sample size to achieve statistical power, provide randomization schemes, etc.), conduct, analysis interpretation, and reporting of clinical, laboratory, and population science studies [103-105]. These tasks may be achieved via performing interim and final data analysis, implementing research databases etc; in addition, biostatisticians may offer short term consulting to researchers during the preparation of research projects/grant proposals or to those who require assistance in the statistical significance of result interpretation. The Biostatistics SR collaborates with an IRB (Ethical Committee) by providing statistical review of each clinical and therapeutic study before Committee assessment of the studies (the presence of a biostatistician among trial investigators may be required in some institutions). Finally, the SR may dedicate part of its activity to the development of new statistical methodology as well as training and educational activities directed toward Cancer Center biomedical investigators.

The establishment of this SR requires adequate space and availability of state-of-the-art computers with programming and statistical software with access to institutional databases. Biostatisticians, who may have sufficient expertise in basic research techniques or in clinical trial design/development, essentially compose the staff.

Many Cancer Centers recognize that biostatistics collaborations are difficult to define in terms of hourly units and that the free exchange of ideas is essential to ensure a fruitful collaboration between the members of the Center. For these reasons, many Institutions do not apply charge back systems to this facility's services. On the contrary, few other Institutions consider the Biostatistics SR similar to other SR and charge back the services provided to investigators.

### Pharmacology

The discovery of anticancer drugs is undergoing a period of rapid changes [106-108]. In fact, the characteristics of new drugs may be completely different from those of traditional antineoplastic drugs. In particular, the pharmacological mechanisms of action of these new drugs are often well-known and include targeting of molecular pathways involved in cancerogenesis and tumor progression [106,108]. For this reason, clinical trials of these new molecules should integrate the traditional measurements (i.e. pharmacokinetics and pharmacodynamics) with molecular analyses (i.e. pharmacogenetics/omics) that are necessary to explain and predict drug safety, the development of resistance mechanisms based on target modulation, and ultimately clinical outcome. In addition, there is increasing evidence that trials under development in selected populations such as aged individuals, who include approximately 60% of all cancer patients, must be designed on the basis of physiologic changes induced by aging that profoundly affect the pharmacokinetics and pharmacodynamics of anticancer therapies [109]. Since pharmacokinetic and biomolecular techniques are particularly complex and technically demanding, the establishment of a Clinical Pharmacology SR is mandatory in Cancer Centers having consistent clinical trial programs.

This SR performs standard methods, develops and validates new assays to perform pharmacokinetic, pharmacodynamic, and pharmacogenetic/omic analyses for clinical and preclinical drug development studies [110]. This SR also provides consultation to researchers in study design involving drug experimentation and develops new methods or uses validate tests for the definition of patient genetic characteristics relative to efficacy or non-response to treatments. In fact, it is now possible to differentiate responders early in drug development; a substantial contribution to afford rapid therapeutic decisions in clinical trials.

Laboratory instrumentation for this SR includes the most sensitive detectors available for the quantitation of analytical molecules; like triple quadrupole mass spectrometers, ion trap spectrometers that can fragment and analyze the mass of compounds, HPLC systems for the separation and analysis of drugs or drug-derived compounds, and equipment for the preparation and storage of biological samples.

Real-time PCR technology, minisequencing reaction by synthesis, high throughput genomic technology platforms, and technologies for proteomic analyses (2D-DIGE, MALDI-TOF mass spectrometry) are particularly important in the development of analytical tools to map genetic loci influencing drug effects or defining the molecular characteristics of drug targets in tumor cells [111,112].

### Clinical research office

The Clinical Research Office (in some Cancer Centers named Clinical Protocol and Data Management, Clinical Trials Office, etc) is a shared resource dedicated to programs of clinical research and provides administrative, scientific, and educational support to clinical investigators through a dedicated staff. Clinical Trial Protocols are reviewed by an Internal Review Board in the U.S. (governed by Title 45 Code of Federal Regulations, part 46) and by an Ethics Committee in the E.U. (Directive 2001/20/EC and further implementations), both of which are regulated by specific laws. These Committees are responsible for protection of human subjects involved in clinical trials and provide public assurance of that protection. The clinical trials approved by the Committees can be initiated and processed, usually by the centralized Clinical Research Office [113-115]. This SR facilitates the development of new clinical trials, supports ongoing clinical trials through centralized data collection, management and reporting, and assures appropriate standards to include quality programs. Internal audits are an essential part of the Clinical Research Office activity. They include controls on eligibility, informed consent, compliance with protocols, adverse events and compliance with Good Clinical Practice and national/international (i.e. European Community) regulatory issues. Besides these optional activities, the SR develops strategic plans for increasing access and accrual to clinical trials, supports relationships with industry, and provides educational programs for clinical researchers [114,116]. This SR requires massive financial investments in human resources, including an additional budget for space and informatics resources. In some Institutes, management software is built in-house, while other Centers use commercially available, web-based systems; the cost of these systems may be relevant, depending on several factors, including the amount and complexity of the data included. Financial support for CRO activity may come from institutional funds, peer-reviewed funding, or pharmaceutical company sponsors. The SR is led by a Medical Director, who is responsible for the activity prioritization, staffing decisions, and for reviewing the clinical trial budgets together with an Administrative director, and for assigning the daily functioning of the SR. The number of personnel employed within this SR depends essentially on its workload and, in bigger Institutes, it may include dozens of persons. Usually the staff of the Clinical Research Office is composed of research nurses, data managers (the most represented numerically), and administrative assistants. Research nurses have clinical responsibilities, such as conducting patient eligibility determination and registration, facilitating the informed consent process, as well as obtaining and delivering biological specimens to the laboratory/biobanking facility. They also have documentation responsibilities, such as accurate submission of patients' data, maintenance of documentation for patient evaluation, participation in auditing, etc. Data managers are responsible for data retrieval and reporting, protocol management, scheduling and hosting audits, including storage and retention of the documents pertaining to research protocols. This activity can be divided into programs; such as breast or lung programs, or else by special areas of investigation such as phase I/II studies. The Clinical Research Office also has solid relationships with the Biostatistics, Bioinformatics, and Biobanking SRs.

### Innovative shared resources

New types of centralized facilities have been recently developed based on complementary innovative approaches. Examples of these types of innovative SRs, are selected in the following:

### Human immune monitoring

This SR is designed to provide advances testing systems to measure immune function in patients, especially when is necessary to evaluate the effects of therapies in patients enrolled in clinical trials. Although correlates of immune protection in infectious diseases may be hypothesized [117], a central problem of human immunology is the lack of markers or correlates that delineate healthy individuals from those affected by various diseases that have a basis in immunological mechanisms [118,119]; in addition, it is becoming clear that results obtained in animal models are often not useful when applied to humans [118]. For these reasons, human immune monitoring facilities prospectively represent an essential resource to advance in the understanding of immunological information that may be incorporated in standard clinical practice. Immune monitoring facilities usually include technologies that in many cancer research centers are part of other distinctive SRs, such as flow cytometers and cell sorters to analyze cells from peripheral blood or lymphoid organs, advanced instrumentation for the multiplex assay of soluble molecules (antibodies, cytokines, soluble receptors, etc) such as the Luminex platform, gene expression microarray systems that are becoming essential to investigate immunological mechanisms in various disease states [120,121] or other genomic technologies to analyze genetic polymorphisms relevant to disease pathogenesis [122,123]. This SR may also engage researchers in order to identify new technological platforms, in vitro or in vivo assays, or bioinformatics procedures that could effectively be used to monitor the immune system under various physiological or pathological conditions. Cellular-based therapies require that source cells be identified, collected, processed, stored, transported, and administered. Therefore, each step must incorporate procedures ensuring the integrity and safety of the final product [124]. For these reasons, when cell-based immunotherapeutic protocols are part of institutional research programs, a Good Manufacturing Practice (GMP) facility may be required, as an additional part of the Immuno-Monitoring facility SR or as a distinct entity when cellular therapy programs encompass many Research Institutes or Universities. In this case, the institution must provide a variable, although usually consistent, investment in space, infrastructure to ensure an appropriate level of environmental cleanliness, instrumentation to process and store biological samples, and well-trained personnel to adhere to the regulatory requirements that are governed by the FDA in the U.S. and by the European Commission in Europe [125,126]. The structural characteristics and space allocated in a GMP facility depend on several factors. Firstly, the type of manipulations that are performed: peripheral blood or bone marrow stem cell transplantation requires minimal manipulations, while "cellular therapies", like infusion of tumor-specific CTLs, require extensive manipulations leading to enhancement of cell proliferation or changes in genotype [127,128]. A second, equally important, conditioning factor is the containment level to be achieved according to the type of microorganisms contaminating cellular products to be manipulated, for example in the case of peripheral blood cell collection from and re-infusion into HIV+ patients.

### Radiation research

Radiation research SRs are built to study the effects of radiations (gamma-rays, x-rays, or UV light) on cellular processes and on cancerogenesis in small animal models. They may provide ancillary services, like assistance with radiobiological data interpretation or irradiation of cell lines or feeder cells, that may not be available to single research groups because of high purchase prices, radiation safety, and regulatory agencies concerns, as well as expertise in the use of radiation sources. These facilities may be equipped with gamma rays or x-ray irradiators for targeting small macromolecules such as DNA or proteins, microorganisms, mammalian cells or small animals. These SRs may be preferentially located in Radiology or Radiotherapy Departments to facilitate their functioning according to national regulations on radiation safety.

The Spatio-Temporal Targeting and Amplification of Radiation Response (STTARR) innovation Center of the University Health Network in Toronto constitutes a remarkable innovative model of radiation research facility [129]. This Center is organized into 4 cores: the cellular core supports genomic and proteomic testing for the prediction of radiation response and toxicity, the Preclinical Core develops investigations on novel radiotherapy strategies in animal models, the Clinical Core is devoted to the development of innovative imaging and treatment in patients, and the computational Core registers and analyzes the data obtained [130]. This Center cannot be merely considered as an institutional SR, since it is based on extensive financial investments to support the building in its location, the impressive qualitative and quantitative variety of instrumentation that is hosted therein and a remarkable number of multidisciplinary researchers who develop research programs. This Center offers unique opportunities to a large number of investigators from the UHN, from other institutions, and from industrial companies new modalities of radiation therapy development. For this reason, it can be considered as a "national" or "international" resource rather than an institutional SR.

### Translational research

Translational research SRs are often laboratory facilities designed to support specific research programs including pre-clinical, experimental phases and/or post-clinical analyses in patients enrolled in clinical trials and maintain databases on sample information. As examples, these SRs may include immunological monitoring laboratories or molecular oncology laboratories in institutions that decided not to develop SRs based solely on these individual technologies. Translational research facilities may also offer consultations for the startup of translational research protocols or promote interactions between basic scientists and clinicians to develop interdisciplinary programs; however, at least in US, the strategies relating to development of effective tools that foster interdisciplinary research and training depend on the Center's Director or the Board of Directors rather than research infrastructure.

## Conclusion

Research infrastructure represents an essential tool in developing successful programs in translational research. Each center needs clear policies on development and on the rules governing the establishment of SRs and the availability of financial resources to set up and maintain these facilities. However, the scientific and economic advantages of an efficient SRs program largely justify the required efforts.

The physical build-up of SRs is, however, not sufficient for the successful translation of biomedical research. Appropriate policies to improve the academic culture for collaboration, the availability of educational programs for translational investigators, the existence of administrative facilitations for translational research and an efficient organization supporting clinical trial recruitment and management represent essential tools in providing solutions to overcome existing barriers to the development of translational research in biomedical research centers.

## Competing interests

The author declares that they have no competing interests.
